# Evaluation of the prevalence of congenital sensorineural deafness in a population of 72 client-owned purebred white cats examined from 2007 to 2021

**DOI:** 10.1186/s12917-022-03378-2

**Published:** 2022-07-22

**Authors:** Kortas Annemarie, Rytel Liliana, Kołecka Małgorzata, Pomianowski Andrzej

**Affiliations:** 1 Department of Internal Diseases with Clinic, Faculty of Veterinary Medicine, University of Warmia and Mazury in Olsztyn, ul. Oczapowskiego 14, 10-719, Olsztyn, Poland; 2 Neurology and Neurosurgery Department, Small Animal Clinic Kalbach, Max-Holder Strasse 37, 60347 Frankfurt, Germany

**Keywords:** Hearing, Hereditary, Feline, Brainstem auditory evoked potentials

## Abstract

**Background:**

Data on sensorineural deafness (CSD) in purebred white client-owned cats is limited as most of the information on this disease entity is assured from mixed-breed experimental colonies. It is known that cats with blue irises are more predisposed to CSD having been described as a condition in which many structures in the inner ear are damaged resulting in hearing loss. Cats with CSD are born deaf or lose their hearing irreversibly within the first 4-5 weeks of life. It is important to diagnose cats with this hereditary condition in order to eliminate affected individuals from breeding. The objectives of this study were to ensure data on prevalence of CSD in a population of 72 client-owned purebred white cats in Poland according to the color of the irises and to determine if there are any predispositions with regard to CSD among different breeds of cats in which the dominant W gene is present.

**Results:**

Conducted study included 72 purebred white cats from six different breeds. The prevalence of CSD in the conducted study was 16.7%, CI_95_ [8.9%; 23.3%]. Unilateral deafness (11.1%, CI_95_ [4.9%; 20.7%]) was more common than bilateral CSD (5.6%, CI_95_ [1.5%; 13.6%]). The studies did not show any association between sex and CSD, *p* = .46. No association between the blue color of irises and deafness in the studied population could be found, *p* = .91. When compared to the rest of the examined population, no association was found between CSD and a particular breed.

**Conclusions:**

Overall prevalence of CSD regarding the examined population of purebred client-owned cats was reported as lower when compared to previous studies concerning purebred cats. Cats with blue irises are more likely to be deaf in accordance to the current state of knowledge, however in the conducted study, no significant association between the presence of blue irises and deafness in white purebred cats could be identified. In order to eliminate CSD from the population, it is necessary to conduct examinations and diagnose CSD in white cats with blue irises as well as with irises of color other than blue. Association between particular breed and CSD wasn’t identified.

## Background

Congenital sensorineural deafness (CSD) in cats has been an area of research for years, however, despite the numerous studies, many aspects of this disease remain unexplained [1]. CSD in white cats with blue eyes was used as a model in studies of human Waardenburg syndrome [2–4]. Nonetheless, as far as it is known, there are only two previously conducted studies that provide data for this condition among client-owned purebred white cats [5, 6]. To our knowledge, this is the first study that provides data for CSD in white cats in Poland.

CSD is particularly prevalent in cat breeds in which the dominant autosomal white gene (W) is present [7, 8]. Described condition is characterized by early postnatal initiation of sensorineural hearing loss [1, 2]. In histological examination, affected cats show pathological changes. The initial step in the pathology is the degeneration of the stria vascularis caused by the loss of melanocytes [9, 10]. One of the primarily observed change which takes place later is the collapse of Reissner’s membrane [9, 10]. Numerous studies have provided evidence exposing lack of melanocytes in stria vascularis linked to CSD. Melanocytes taking their origin from neural crest are comprised in the stria vascularis [11–13]. This blood rich zone takes responsibility for the assurance of the endocochlear potential through liberation into the endolymph high levels of K + , which is necessary for activating electrical potential in the hair cells [12, 14].

Genetic mapping research has shown an association between white spotting phenotype and the KIT gene on feline chromosome B1 [15]. Among many functions of the KIT, the effect of its stimulation on melanocytes has also been demonstrated [16–18]. According to recent reports, white coat phenotype in cats is associated with a mutation involving partial insertion of the genetic material of the feline endogenous retrovirus (*FERV1*) in the KIT gene. The study has shown that partial insertion of the FERV1 could be also associated with the occurrence of CSD among white cats [19, 20].

To exclude cats with CSD from further breeding, the determination of hearing using brainstem auditory evoked responses (*BAER*) is a procedure often performed in veterinary clinics. This exclusion is an attempt to eliminate the genes responsible for CSD from populating, even though the exact mechanisms of inheritance have not been discovered despite numerous studies [7]. Prior to the two studies conducted independently, in which the examined populations of client-owned cats differ [5, 6], data were collected from experimental colonies and are not credible when it comes to client-owned cats due to disparate breeding concepts. The limited prevalence data regarding client-owned cats and the lack of clearly defined breed predispositions justify the necessity for further research among purebred client-owned cats.

The objectives of this research were to provide data on prevalence of CSD among a population of 72 purebred client-owned white cats in Poland by taking the color of the irises into consideration and to determine CSD prevalence among different breeds of cats in which the dominant W gene is present.

## Results

Seventy-two purebred white cats among six different cat breeds were included in the study. Feline patients were 27 Maine Coons, 23 Norwegian Forrest Cats, 8 Sphynx, 7 British Shorthairs, 6 Devon Rexes, 1 Cornish Rex.

The overall prevalence of CSD in the studied population was estimated at 16.7% (12/72), CI_95_ [8.9%; 23.3%]. The percentage regarding unilaterally deaf cats was 11.1% (8/72), CI_95_ [4.9%; 20.7%], and the remaining 5.6% (4/72), CI_95_ [1.5%; 13.6%] of cats were bilaterally deaf. All female cats that were affected with CSD were unilaterally deaf.

The examined subjects involved 32 male and 40 female cats. The differences regarding prevalence of CSD between male and female cats were not significant χ2 (1, *N* = 72) = 0.55, *p* = 0.46. The median age of all patients was 15 weeks ranging from 8 to 288 weeks.

The data regarding the iris color were confirmed in all 72 cats. Out of 12 cats with both blue irises, 2 cats were affected by CSD (16.7%), CI_95_ [2.0%; 48.4%], among 14 cats with one blue iris, 2 cats were affected by CSD (14.3%), CI_95_ [1.8%; 42.8%], and among the population of 46 cats with no blue iris, 8 cats were defined as affected by the described condition (17.4%), CI_95_ [7.8%; 31.4%]. In the studied population, there was no significant finding regarding statistics that cats with at least one blue iris were more likely to be deaf than cats whose irises were colors other than blue χ2(1, *N* = 72) = 0.01, *p* = 0.91. Difference in CSD prevalence between cats with 1 or 2 blue irises was not significant either χ2 (1, *N* = 72) = 0.14, *p* = 0.70. Out of 14 animals having one blue iris, 2 cats were unilaterally deaf (14.3%), CI_95_ [1.8%; 42.8%], and the deafness sides of both individuals matched to the side of the single blue iris. Within the breeds represented by the largest number of subjects: Maine Coon (27) and Norwegian Forest (23), no differences in the prevalence of CSD were found between individuals with at least one blue iris and individuals without a single blue iris (Maine Coon: χ2 (1, *N* = 27) = 0.14, *p* = 0.71; Norwegian Forest: χ2 (1, *N* = 23) = 0.35, *p* = 0.55). The number of cats examined specifying the color of the iris and breed is presented in Table 1.

**Table 1:** Hearing status of the examined cats

Breed	Number of cats	Cats with 2 blue irises	Cats with 1 blue iris	Cats with 0 blue irises	CSD	CSD in cats with at least 1 blue iris	CSD in cats with none blue iris
Maine Coon	27	3	5	19	4/27	1	3
Norwegian Forrest	23	5	3	15	3/23	1	2
Sphynx	8	2	4	2	2/8	2	0
British Shorthair	7	0	0	7	2/7	0	2
Devon Rex	6	1	2	3	1/6	0	1
Cornish Rex	1	1	0	0	0/1	0	0

While comparing cats from a particular breed to the remaining examined individuals, no significant association with related to CSD was found for Maine Coon and Norwegian Forrest breed (Maine Coon *p* = 1.00; Norwegian Forrest χ2 (1, *N* = 72) = 0.05, *p* = 0.82). Small sample sizes regard to Sphynx, British Shorthair and Devon Rex prevented determining whether significant associations between these particular breeds and CSD were seen. Cornish Rex breed was excluded from the comparison because it was represented just by single individual.

In order to extend the statistical analyzes, univariate logistic regression was performed. Obtained results did not reveal any significant predictors for CSD (p > 0.05 for all variables: breed, sex and number of blue eyes), which is presented in Table 2.

**Table 2:** Univariate logistic regression for CSD

Predictor	OR	95% CI for OR	*P*
Breed (British Shorthair = reference)			
Cornish Rex	0.01	n/a	.992
Devon Rex	0.50	0.02 to 7.01	.615
Maine Coon	0.43	0.06 to 3.75	.403
Norwegian Forrest	0.38	0.05 to 3.42	.346
Sphynx	0.83	0.08 to 9.16	.876
Sex, male	1.96	0.56 to 7.30	.294
Number of blue eyes (0 = reference)			
One	0.79	0.11 to 3.72	.785
Two	0.95	0.13 to 4.58	.9 53

*OR* odds ratio, *CI* confidence interval

## Discussion

For a long time, researchers have been focusing on the subject regarding the deafness in white-coated cats with blue irises [2–4, 7]. The preceding research focused mainly on mixed breed experimental colonies which were used as a model in studies of human Waardenburg syndrome [2–4]. Further, prevalence data from previously conducted studies on client-owned purebred white cats are assured [5, 6]. The prevalence of deafness significantly differed in cats belonging to mixed breed colonies compared to client-owned cats. Moreover, the prevalence of CSD in purebred white client-owned cats obtained in two independent studies differed significantly, and was estimated as follows 20.2% [5] and 30.3% [6].

The objective of the research was to determine the prevalence of CSD in a population of client-owned white cats, and as to whether there are significant differences in the prevalence of CDS regarding the color of the irises in the studied population. Additionally, the goal was to compare the prevalence of this disease entity within the studied breeds in order to determine as to whether there are breed predispositions that characterize individual breeds. To our knowledge, this is the first data describing a population of purebred white cats in terms of CSD in Poland.

Seventy-two purebred white cats were tested, and the overall prevalence of CSD was 16.7%. By comparing the prevalence outcome to the data obtained in the previous studies regarding client-owned cats, this study reports lower prevalence rates [5, 6]. In previously conducted studies, cats were stimulated with the intensity of 70 or 80 dB normal hearing level [5], and with the intensity of 80 dB [6]. In both aforementioned studies that were previously conducted, when no response was noted, the intensity of stimuli was increased to 90 dB and 100 dB respectively. It should be noted that there were slight differences in prevalence when compared to the occurrence rate of 20.2% [5] but these differences became even more contrasting while comparing to data introduced by Mari et al. [6] in which the overall prevalence was 30.3%. These differences may result from the disparate reproductive strategies and a different genetic pool characterizing the above-mentioned populations. They may also result from the dissimilarity of the studied populations in terms of the number of cats belonging to particular breed and the overall number of cats included in studies. Dissimilarities in obtained the data may also result from different criteria for the inclusion of cats in the studies, however, to our knowledge, there are no other studies that are conducted on purebred white cats. The lack of a sufficient number of reports describing especially purebred cats indicates the need to continue research in order to determine the prevalence of sensorineural deafness.

Norwegian Forest cats were found to be significantly associated with CSD when compared to non-Norwegian Forest breeds gathered together, and constituted the highest percentage of population (< 32%) in the examined group of cats [6]. Norwegian Forest cats in the examined population in Poland constituted 31.9% of all examined cats, and despite a slight difference regarding percentage value compared to the previous research, it was not possible to discover any statistically significant association that characterizes this particular breed (*p* = 0.74). In contrast to the data presented by Mari et al. [6] regarding the Norwegian Forest Cats, it is impossible to compare the obtained data with the data presented by Cjevic et al. [5] regarding Norwegian Forest cats because the number of cats in examined population was significantly lower (> 8%). Even though the conducted research failed to present breed predispositions within any of the studied breeds, which is not the case of the Norwegian Forrest breed in the previous study [6], more studies with different study designs and more examined individuals are needed. Despite the fact that a small number of cats representing the Sphynx breed made it impossible to establish the existence of an association between CSD and this particular breed, a particular attention was decided to be paid to this breed. Prevalence of deafness in the studied Sphynx population was 25%, and deafness was only present in cats with at least one blue iris which is consistent with the current state of knowledge indicating the predisposition of blue-eyed cats to CSD. The study conducted by Cjevic et al. [5] have not included this particular breed, and the other study [6] didn’t establish the prevalence of deafness for this particular breed. Lack of any previous data indicates the necessity for further research describing the prevalence of CSD among this individual breed. The highest number of cats included in the conducted study belonged to Maine Coon breed, and were accounted for 37.5% (27/72) of the cats tested. The overall prevalence of CSD among this breed was < 15%, which is a noteworthy frequency when compared to the results obtained in foregoing studies in which the prevalence of CSD among this individual breed was as follows < 35% [5] and < 44% [6]. The indicated difference may result from the different number of tested cats representing this breed in the compared research groups. It may also be an outcome of the fact that all three compared study groups consisted of client-owned cats which have undergone clinical trials in which it is difficult to obtain homogeneous research groups. The differences in the prevalence of CSD were also noted with regard to the British Shorthair, which in the study was one of the breeds represented by the least number of individuals, whereas it was the largest group of the studied individuals in data presented by Cjevic et al. [5]. Hence, differences in prevalence can be justified by analogous relationships mentioned in the case of the Maine Coon breed. In the previous studies, the number of Devon Rex solid white cats was 1 [5] and 12 [6], respectively. Conducted study included only 6 representatives of this breed. Disproportions in the groups consisting of few individuals clearly indicate the need for studies that would cover larger populations. Additionally, while searching for associations between particular breeds of cats and CSD, statistical analyzes were extended, and univariate logistic regression was performed, however, as in the case of analyzes performed with the use of the Yates’s Chi-Squared test, no associations were found between any of the breeds presented in the study and CSD. The detailed results of the conducted analysis are presented in Table 2.

Difficulties in examining a sufficiently large number of individuals during clinical trials could be resolved through the cooperation of more centers involved in the study of deafness in cats, based on the exchange of data regarding study results. Undertaking such cooperation could make it possible to determine the prevalence of CSD in particular breeds of purebred cats and it would also allow to determine the breed-specific predispositions.

Obtained data exhibit that unilateral deafness was more common in the conducted research. The occurrence of unilateral and bilateral deafness was as follows 11.1% and 5.6%. It is worth to mention that all females that were affected with CSD were unilaterally deaf. In the previous studies, the differences in the incidence of unilateral and bilateral deafness were insignificant. However, it should be emphasized that in both previous studies, bilateral deafness was more frequent [5, 6]. This difference may be due to the specifics of clinical trials; however, it requires further evaluation by the studies that will be conducted in the future.

Results obtained in conducted study do not show any significant association in the prevalence of CSD and the sex χ2 (1, *N* = 72) = 0.55, *p* = 0.46, which is consistent according to previous reports [5, 6]. Moreover, parallel to the Yates’s Chi-Squared test and in accordance with the previous studies [5, 6], while performing univariate logistic regression, no associations between the sex of cats and CSD were found (OR = 1.96 CI_95_ [0.56;7.30] *p* = 0.294) (Table 2).

The differences in the acquired outcomes in regards to the prevalence of deafness as per the color of the irises comparable to the previous studies are particularly noteworthy. In previous studies, it was agreed that cats with at least one blue iris are more predisposed to CSD compared to the cats without blue irises at all (*p* = 0.04 [5]; χ2 = 0.01, *p* = 0.91 [6]), which is in line with the current state of available knowledge. In the conducted study, an unexpected data was obtained which indicates that in terms of statistics, there is no significant association between the blue color of the irises and deafness χ2(1, *N* = 72) = 0.01, *p* = 0.91 in the examined population of cats. In the univariate logistic regression, no associations were found between the presence of one (OR = 0.79 CI_95_ [0.11;3.72] *p* = 0.785) or two (OR = 0.95 CI_95_ [0.13;4.58] *p* = 0.953) blue irises and the presence of CSD. The outcome of the results may be the consequence of several specific factors describing the studied population. First of all, the major population of this conducted research consisted of cats with both irises of a color other than blue and accounted these cats as > 63% (46/72) of the cat population included in the statistics. Obtaining an outcome inconsistent with the previous reports may result from the fact that the remaining populations of the client-owned cats examined differed significantly from the population included in the study. In the first independent study, the percentage of cats with at least one blue iris was > 50% [5], while the subjected population in Poland consisted of < 37% (26/72) cats having at least one blue iris. When the subjected population with the population studied in another independent research is compared [6], the differences in the percentage of cats with at least one blue iris in the studied populations are similar, and the percentages are as follows > 33% and > 37% respectively. However, it should be noted that the cat population included in the previous study exceeds the number of cats included in the hereby conducted study, and thus the number of cats with at least one blue iris exceeded the number of these particular cats included in the examined population in Poland. It should be noted that the largest group represented in the study by Maine Coon cats consisted of > 30% of cats with one blue iris, whereas in the compared population of cats the percentage of Maine Coons with one blue iris was > 61% [6]. With regard to the Maine Coon breed, it should be additionally noted that 75% of deaf cats within this breed had both irises of a color other than blue.

In the research, among 14 cats with one blue iris, two cats were deaf and both individuals were unilaterally deaf, and the side of the blue eye matched the side of deafness. A similar relationship was found in the previous studies in which it was found that in 50% of the side of unilateral deafness matched the side of single blue iris [6]. The obtained observation requires further research on a larger population of cats with one blue iris, which would allow to determine the existence of potential association.

The recorded disproportions distinguishing the conducted study from previous studies on client-owned cats might have an impact on the unexpected outcome that the presence of at least one blue iris is not significantly associated with CSD in terms of statistics. The differences in the obtained data may result from the difficulty in acquisition of identical research groups, which characterizes clinical trials. The fact is that the breeders of purebred cats are aware of the generally accepted knowledge about the predisposition of white cats with blue irises to CSD, and despite choosing reproductive strategies limiting the number of blue-eyed cats in white litters, sensorineural deafness still appears in white cats with irises other than blue. Therefore, it should be concluded that not all purebred white cats with blue irises are deaf, and that CSD appears in white cats with irises other than blue. During clinical trials it appears that breeders of white cats with irises other than blue are surprised by the obtained result of the study confirming deafness in the cat. This type of situation clearly indicates the need to increase the amount of research on the pedigree cat population in response to the growing interest of breeders and owners. In order to eliminate sensorineural deafness from the population, it is necessary to conduct tests and diagnose CSD in white cats with blue irises as well as with irises other than blue. Ultimately, the mechanisms of inheritance are yet not explained [7], which makes it impossible to exclude the presence of possibly occurring unknown factors that would unequivocally justify the difference of the obtained results.

## Conclusion

To sum up, the overall prevalence was lower than previously conducted studies regarding client-owned cats and was estimated as 16.7%, which suggests a lower prevalence of CSD in the examined population in Poland. The obtained data specifically defining the studied population, which differ from previous reports on client owned cats [5, 6], show no association between the presence of blue irises and deafness. Even though a particular attention on possible breed association was paid, it was not possible to discover the relationships sought. Mentioned differences may be due to a different gene pool in the studied populations, however, obtained results indicate the necessity to conduct further studies involving white cats with blue irises and other color of irises, and justify the validity of conducting further studies focusing on individual breeds of cats. The interest to eliminate CSD from the purebred cats population in Poland is confirmed by the resolution of the General Meeting of Members of the Polish Felinological Federation,,Felis Polonia’’ (FPL), effective from 01/01/2021, requiring BAER testing in white cats intended for breeding [21]. Mentioned resolution legitimizes the growing interest to eliminate CSD from the population of purebred white cats in Poland and the increasing demand for the necessary data regarding the implementation of the screening process.

## Methods

### Animals

As part of a screening program to exclude deaf individuals from breeding, a study was conducted between April 2007 and February 2021, which included a total of 72 white purebred cats which were the patients at the Department and Clinic of Internal Diseases of the Faculty of Veterinary Medicine of the University of Warmia and Masuria in Olsztyn, Poland. Cats were examined retrospectively till the September of 2020, as of this date cats were examined prospectively. A detailed interview with animals’ owners and basic clinical and neurological examination were performed in all cats before examining them with brainstem auditory evoked responses [22]. Data files of the examined cats included breed, age, sex, coat color, iris color, and electrodiagnostic specification estimated with brainstem auditory evoked responses. Recordings were performed in a quiet room without soundproofing. All data was obtained in accordance with the principles of good clinical practice; however, ethics approval was not required during the period of data collection. Pursuant to the law in force in Poland, activities performed as part of a veterinary practice do not require the consent of the Ethics Committee. Cats included in the study had to meet the following criteria: purebred white cats at least 8 weeks old; non-objective physical and neurological examination result; no history of topical administration of potentially ototoxic drugs; no history of ear disease [23].

### Methods

**Fig. 1:**
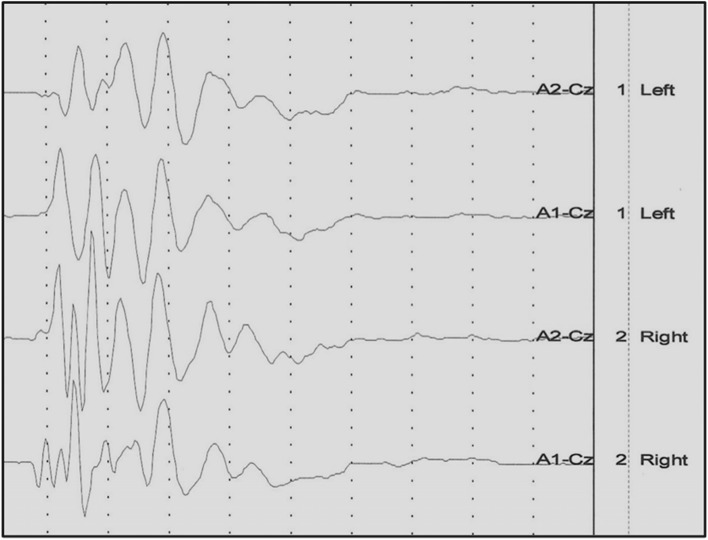
The result of the BAER examination of a cat with normal hearing in both ears

**Fig. 2:**
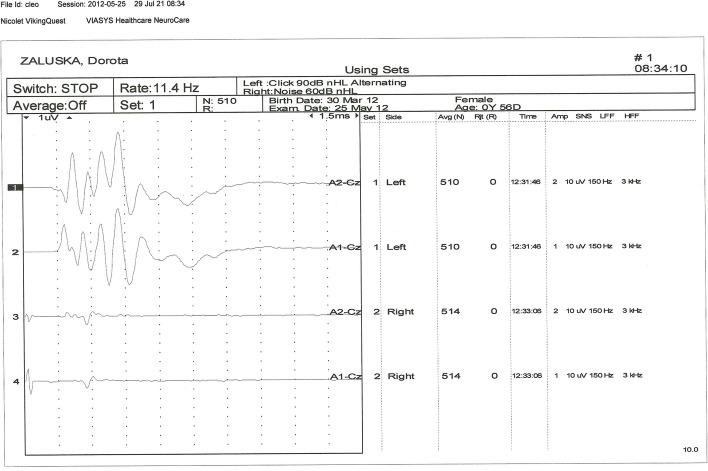
The result of the BAER examination of a cat with unilateral deafness

**Fig. 3:**
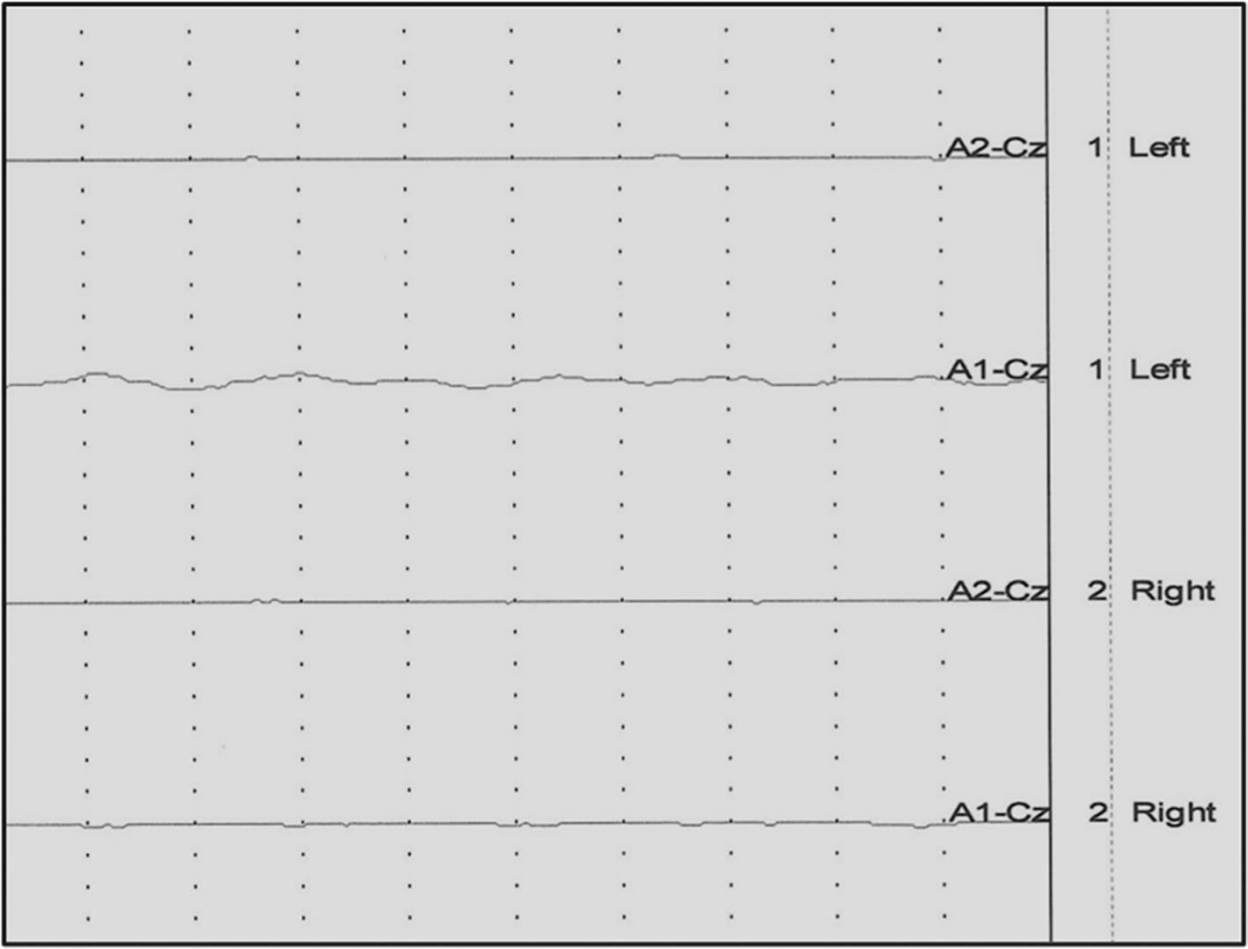
The result of the BAEP examination of a cat with bilateral deafness

After the results of basic clinical and neurological examination having shown no contraindications for sedation, all tested animals were premedicated with medetomidine hydrochloride (Domitor R, Orion Corporacion Orionintie 1FIN—02200 Espoo Finland) at a dose of 50 – 150 μg/kg of body weight by intramuscular injection into the left or right quadriceps muscle. For the recordings, cats were placed in sternal recumbency on a special wooden table equipped with a rubber insulating mat. Insert earphones adjusted to the diameter of the external auditory canal were introduced in the external auditory canal, as close as possible to the transition of the vertical to the horizontal part [24]. Four subcutaneous, disposable, steel needle electrodes (VIASYS Healthcare, Neurocare Group, Madison, WI 53711–4495 USA) were placed in the following configuration: recording electrode at point Cz (at the top of the dome of the head); reference electrodes at the level of the mastoid processes of the temporal bones on the left and right side; ground electrode on the middle of the neck [7, 24]. In all cats, BAER testing was performed first in the left ear then in the right ear. BAER determinations were carried out on all cats using the same electrodiagnostic unit Viking Quest (Nicolet Biomedical Inc, WI, USA)**.** BAER results were acquired by averaging 500 recordings of 10 ms. Filters were set at the cutoff frequencies of 100 Hz and 3000 Hz, and the signal was amplified 200,000 times [25]. Cats were stimulated with click intensity at 90 dB nHL (dB) in the examined ear. While the stimuli in the form of clicks were being emitted to the examined ear, a masking signal was sent to the opposite ear in the form of the so called “white noise” whose value was 30 dB nHL (dB) lower in order to prevent crossover effect. The animal was classified as normal when there was response from both ears (Fig. 1), unilaterally deaf when there was a response from only one ear (Fig. 2), and bilaterally deaf when there was no response from both ears (Fig. 3). The recordings were concluded and subsequently atipamezole at a dose of of 10 μg/kg i.m (Antisedan 5 mg/1 ml, Orion Corporation Orionintie 1 FIN02200 Espoo Finland) was used to reverse the sedation.

### Statistical analysis

Statistical analysis (Statistica program StatSoft Inc, Tulsa, OK, USA and R: Language and environment for statistical computing by R Foundation for Statistical Computing, Vienna, Austria) were performed using Yates’s Chi-Squared test and logistic regression analysis. All statistical analyses were performed with the level of significance set as p < 0.05. All statistical analyses were obtained with the support of a professional statistician. Yates’s Chi-Squared test was used to determine the association between prevalence of CSD and color of the irises and, additionally, the significance between prevalence of CSD and sex of examined cats. Association between CSD and the individual breeds, when compared to the rest of the examined population, were also estimated with the use of Yates’ Chi-Squared test. The same test was also used to find the differences in association of CSD between individuals with at least one blue iris and individuals without a single blue iris within the two most numerous groups of cats: Maine Coon (27) and Norwegian Forest (23). Prevalence rates regarding bilateral, unilateral and consolidated bilateral and unilateral deafness were estimated. Additionally, binomial exact 95% confidence interval for proportions was calculated as appropriate. Univariate logistic regression analysis was carried out to identify potential predictors of CSD.

## Data Availability

The datasets used and/or analysed during the current study are available from the corresponding author on reasonable request.

## Abbreviations

BAEP: Brainstem auditory evoked potentials
CSD: Congenital sensorineural deafness
FERV1: Feline endogenous retrovirus
